# A Review of Pesticide Residues in Pears: Current Status, Dissipation Pattern and Detection Methods

**DOI:** 10.3390/foods14091470

**Published:** 2025-04-23

**Authors:** Li Zhang, Tengfei Liu, Linlin Shi, Libin Wang, Daifeng Yang

**Affiliations:** 1 Center for Food Safety and Nutrition, Suzhou Vocational University, Suzhou 215104, China; zhangli_szd@163.com; 2 Jiangsu Taihu Area Institute of Agricultural Sciences, Suzhou 215106, China; 3 College of Food Science and Technology, Nanjing Agricultural University, Nanjing 210095, China; 4 College of Horticulture, Nanjing Agricultural University, Nanjing 210095, China

**Keywords:** pear, pesticide residue, current status, dissipation pattern, detection method

## Abstract

Pears are pome fruits widely cultivated and consumed globally due to their unique taste and flavor. The application of pesticides is a significant practice to combat pear pests and diseases across the world; however, pesticide use during production frequently results in undesirable residues in postharvest pear products, raising concerns about potential health impacts. Thus, the monitoring of pesticide residues (PRs) in pears has always been of great importance. To assess the degree of PRs in pears, sensitive and selective techniques are required to detect them. This review article attempts to provide readers with an overview of analytical methods utilized for the quantitative analysis of PRs in pears. Sample preparation and detection techniques are described and compared, and the advantages and disadvantages of these procedures are discussed. Furthermore, this paper elucidates the occurrence characteristics and dissipation patterns of PRs in pears through a comprehensive analysis of global research data on pear crops. In addition, potential trends for future research are presented, with the goal of contributing valuable insights to ongoing studies on PRs in pears and other fruits.

## 1. Introduction

**Figure 1:**
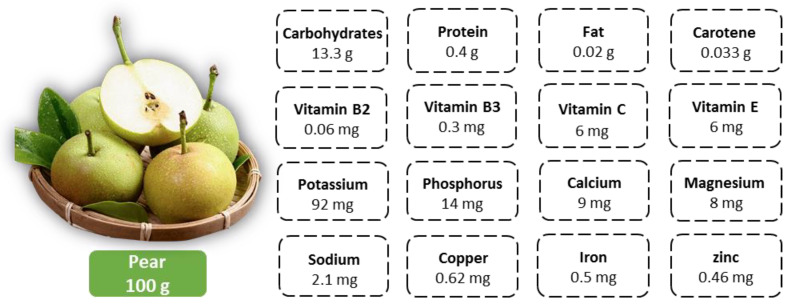
Mean nutritional content per 100 g of pear.

Pear (*Pyrus* spp.) is a perennial woody plant of the *Pyrus* genus in the Rosaceae family and ranks among the most widely grown pome fruits worldwide [1]. Characterized by its crisp texture and aromatic flavor, the pear is also a rich source of essential nutrients (e.g., protein, vitamins and minerals) [2,3], as detailed in Figure 1. These attributes have significantly contributed to their widespread consumer appeal. As reported by the U.S. Department of Agriculture (USDA), global pear consumption reached 25.76 million tons in 2024 [4]. China is the world’s leading pear producer, with a cultivation history over 3000 years and a wealth of genetic resources [5]. In recent years, China’s pear industry has witnessed steady development, with both the harvested area and yield ranking first in the world. According to the FAO, by the end of 2022, China’s pear cultivation area had expanded to approximately 1.005 million hectares, with a total production of 19.367 million tons, accounting for 70.9% and 73.6% of the global area and production, respectively [6]. China also plays a significant role in the world’s pear market as both a major exporter and consumer. In 2024, China exported 660,000 tons of pears, while domestic consumption reached 19.555 million tons, representing 34.4% and 75.9% of the world’s total pear exports and consumption, respectively [4]. The pear industry has thus become a cornerstone of agricultural development in China, making substantial contributions to poverty alleviation and rural revitalization.

Pears, like other pome fruits such as apples and loquats, thrive in warm, humid and well-lit environments. However, such climatic conditions are also conducive to the proliferation of pests and diseases, which can exert deleterious effects on pear cultivation, including stunted growth, reduced yield and diminished fruit quality. To date, a total of 26 diseases and 62 insect pests have been identified as significant threats to pear production across the globe [7]. To mitigate these biotic stressors, pesticides are intensively applied in pear orchards as a common agricultural practice [8,9,10]. This chemical-dependent strategy, however, has sparked considerable concerns regarding pesticide residues (PRs) in pears. In response, many nations have implemented regulatory measures, such as establishing maximum residue limits (MRLs) [11,12,13,14] and conducting routine monitoring programs [15,16], to regulate and trace pesticide levels. Moreover, extensive research has been carried out to explore residue levels and dissipation patterns of these chemicals. In this context, different techniques have been developed and evaluated for the quantification of PRs, among which chromatography and mass spectrometry are the most prevalently employed, while some other technologies such as immunoassays and sensors are also utilized. Given that PRs in pear samples often occur at low and fluctuating levels, analytical methods applied for their detection are continuously advancing to achieve enhanced sensitivity and efficiency, thereby facilitating real-time monitoring. To our knowledge, a comprehensive and up-to-date overview of this topic remains lacking. Therefore, this review endeavors to present the current knowledge on the occurrence, dissipation and detection methods for the analysis of PRs in pears, in order to provide a valuable reference for monitoring and in-depth exploration of pesticides in pears.

## 2. Occurrence of Pesticides in Pears

The application of pesticides in pear cultivation has become an indispensable practice; however, this has also elevated the issue of PRs to a matter of significant concern. Governments and researchers worldwide have conducted extensive investigations into the occurrence of PRs in pear products. Nevertheless, discrepancies in research focus, scope, methodologies and perspectives have led to varying findings regarding residue profiles. Recent scientific studies and regulatory monitoring programs have revealed several critical characteristics of PRs in pears, providing new insights into their residual patterns.

### 2.1. PRs in Pears Are Prevalent, with Multiple Pesticides Frequently Co-Occurring

The presence of PRs in pears has been occurring for years due to their extensive use in agriculture practices, which has led to their frequent detection in pear samples. A recent study by Eissa et al. [17] analyzed data from the Rapid Alert System for Food and Feed (RASFF) on pesticide notifications in fruits and vegetables (F&Vs) between 1999 and 2022, with the aim of identifying the most frequently reported F&Vs, pesticides and their countries of origin. The study revealed that pears ranked among the top 15 F&V categories affected by PRs, with an overall detection rate of 5.69%. Over this 24-year period, pears received 123 pesticide residue notifications, predominantly from Turkey (57 notifications) and Italy (18 notifications), highlighting the need for ongoing government monitoring to ensure food safety for the population. Furthermore, the most frequently detected pesticides were amitraz (55 notifications) and chlormequat (5 notifications). In another study, Jardim et al. [18] investigated the results of the two Brazilian national pesticide residue monitoring programs conducted between 2010 and 2020. A total of 35,321 samples from 47 different food items were analyzed, revealing that 55.3% tested positive for at least one pesticide. Notably, over 90% of the analyzed pear, peach, strawberry and sweet pepper samples were found to contain residues. Of particular concern, PRs were detected in 97.1% of pear samples, with over 80% containing two or more pesticides. The most prevalent pesticides identified were dithiocarbamates (26.1%), triazoles (19.4%), organophosphorus (11.6%), pyrethroids (10.5%) and N-methyl carbamates (0.5%). The presence of multiple PRs in one single sample can be attributed to the application of different types of pesticides (e.g., herbicides, fungicides, or insecticides) or multiple pesticides of the same type (e.g., different fungicides), as well as poor agricultural practices. According to the USDA Pesticide Data Program (PDP) [16], in 2022, the majority of fresh pear samples in U.S. markets contained detectable PRs, with 91.5% testing positive for at least one pesticide and 84.7% showing detectable levels of two or more pesticides. Strikingly, one sample exhibited residues of 18 different pesticides, underscoring the complexity and extent of pesticide presence in pears. Similarly, the European Union (EU) reports on PRs in food (2020–2022), provided by the European Food Safety Authority (EFSA), demonstrated a high prevalence of PRs in food samples from the European market [19,20,21]. Over the three-year period, the overall detection rates of PRs were 31.5% (2020), 44.3% (2021) and 41.1% (2022), with the detection rates of samples containing multiple residues at 18%, 26.4% and 23.0%, respectively. In particular, pears and six other fruits (peach, orange, apple, strawberry, table grape and sweet pepper) were the unprocessed products with the highest frequency of multiple residues across all three years. The EFSA recommends continued monitoring of these foodstuffs. To evaluate the residue levels of highly toxic pesticides (HTPs) in F&Vs of China, Li et al. [22] conducted a comprehensive survey on HTPs in 6, 554 F&V samples collected from 31 regions across the country between 2014 and 2017. The findings indicated that 18 HTPs were detected in 325 (4.96%) pear samples, with 103 (1.57%) samples exceeding China’s MRLs. Notably, HTPs were detected in pear samples from 15 regions, with the highest detection rate observed in Shandong (2.9%) and the highest exceedance rate in Jiangxi (21.1%). The presence of HTPs exceeding the MRLs in some samples, including pears, highlights the urgent need for implementing stricter management guidelines to safeguard consumer health. Furthermore, a study on PRs in F&Vs from Huili, Sichuan Province, China, conducted between 2020 and 2021, reported a detection rate of 28.6% in pear samples, with cypermethrin, imidacloprid and carbendazim identified as the predominant pesticides [23]. In contrast, another study revealed a significantly higher detection rate of 96.7%, with chlorpyrifos (93.3%) and profenofos (16.7%) being the primary pesticides detected [24]. Moreover, a monitoring study on PRs in fruits from Shaanxi, China, conducted between 2018 and 2021, found that pears were among the fruits with elevated pesticide residue levels among the 15 analyzed. Of particular concern was the detection rate of fungicides, with some samples exhibiting the presence of more than three PRs concurrently [25]. These findings indicate significant regional variability in pesticide residue levels in pears.

In addition, significant differences in PRs have been documented even among pears from the same geographical region. As an example, a study conducted by Chi et al. [26] on 100 pear samples from Jinan, Shandong Province, China, uncovered a detection rate of 31%, with the primary pesticides identified as imidacloprid, acetamiprid, buprofezin, trifloxystrobin and oxyfluorfen. In contrast, Lu et al. [27] reported a lower detection rate of 18%, with pyrethroid pesticides, including fenpropathrin, bifenthrin, cypermethrin and fenvalerate, being the most prevalent. Interestingly, Zhang et al. [28] found a significantly reduced detection rate of only 1%, with malathion identified as the sole pesticide. These discrepancies are likely attributable to variations in detection methodologies and the specific pesticides examined across these studies.

### 2.2. Occurrences of Pesticide Residue Levels Exceeding Regulatory Limits Are Common in Pears, with a Low Overall Exceedance Rate

Scientific consensus establishes that pesticide residue concentrations in food crops are determined by multiple factors. These mainly include the following: (i) the intrinsic physicochemical properties of the pesticides; (ii) the parameters related to field application of pesticides, such as the time and frequency of their usage; (iii) post-harvest processing and preservation techniques; (iv) the sensitivity and specificity of analytical detection protocols; and (v) the regulatory framework of MRLs set by different national or regional authorities [29,30,31,32]. A meta-analysis and systematic review conducted by Ahmadi et al. [33] evaluated the residue concentrations of different pesticides (including insecticide, fungicide, herbicide, acaricide, ovacide, nematicide, miticide and veterinary substances) in global fruits from 1995 to 2021. The findings revealed that among the 27 studied fruits, pears exhibited the highest levels of insecticide residues, with an average concentration of 0.8 mg/kg. In the United States, data from the USDA PDP, which systematically monitors PRs in foods sold in supermarkets, indicated that 0.14% of pear samples had residues above the MRLs in 2021 [15]. Similarly, monitoring data from the EU reports on PRs in food revealed that 5.1%, 3.9% and 3.7% of the tested samples from European markets exceeded the MRLs in 2020, 2021 and 2022, respectively. Notably, pear samples showed an MRL exceedance rate of 2.3% in 2021 [19,20,21]. In contrast, a nationwide survey of PRs in F&Vs across 31 provinces, autonomous regions and municipalities from 2014 to 2017 found that only 0.27% of the 1122 pear samples contained residues exceeding the MRLs. These pesticides were primarily omethoate and phorate, with maximum residue concentrations of 0.0461 mg/kg and 0.0264 mg/kg, respectively [22]. Further regional studies in China have provided additional insights. A survey of PRs in the main fruits (watermelon, grape, pear and mulberry) from Daxing district, Beijing, between 2017 and 2019, revealed that 0.67% of pear samples exceeded the MRL standard for isazophos [34]. Similarly, an investigation into PRs in four characteristic fruits (Miaoxi yellow peach, Lanxi loquat, Qingyuan sweet spring tangelo and Haining pear) from Zhejiang Province between 2020 and 2021 showed that 4.76% of pear samples exceeded MRLs for prochloraz [35]. In Chongqing, a 2021 study observed that 1.4% of pear samples exceeded MRLs for isazophos [36]. In Shandong Province, a surveillance report from 2019 to 2023 identified exceedances of MRLs for carbendazim and emamectin benzoate in pears [37]. Additionally, during 2021–2022, pear samples from planting bases in Yanbian Prefecture, Jilin Province, were found to exceed MRLs for profenofos, cyhalothrin and deltamethrin [38]. In Zhengzhou, Henan Province, 20% of pear samples available for sale exceeded MRLs for deltamethrin and imidacloprid [39]. The above results demonstrate that a percentage of pear samples contained PRs exceeding regulatory limits; however, this does not automatically indicate an actual health risk to consumers [22,35]. The potential health impacts of pesticide exposure depend not only on residue levels but also on multiple other factors, such as the consumption of pears, the toxicological reference values of the pesticides (e.g., acceptable daily intake (ADI) and acute reference dose (ARfD)), as well as individual differences among consumers (e.g., age and body weight) [40].

### 2.3. Multiple Residual Pesticides Occur in Pears, with a Majority Being Unregistered Varieties

According to the USDA PDP, in 2021, a total of 25 residual pesticides were identified in pear samples, with pyrimethanil concentrations notably exceeding the MRL standard [15]. In China, monitoring of PRs in F&Vs conducted by market supervision departments across various provinces (cities and districts) from 2021 to 2022 revealed that multiple residual pesticides in pears exceeded regulatory limits. The primary pesticides of concern included cyhalothrin, lambda-cyhalothrin, carbendazim, imidacloprid, dichlorvos and omethoate [41]. A similar scenario was observed in characteristic fruits from Zhejiang Province, China, between 2020 and 2021, where a total of 16 pesticides were detected in pear samples. The pesticides with the highest detection rates were pyraclostrobin (85.71%), chlorantraniliprole (71.43%), carbendazim (42.86%), chlorfluazuron (42.86%), acetamiprid (33.33%) and lambda-cyhalothrin (33.33%). In a related study, Lan et al. [42] investigated the residual levels of 102 pesticides in pears and apples from Shandong Province, China, between 2014 and 2015. The study identified a total of 37 pesticides in pear samples, including 21 insecticides, 13 fungicides and 3 acaricides. Using the risk ranking matrix of veterinary drug residues of the UK Veterinary Drug Residue Committee, the pesticide risks were evaluated based on six indicators: pesticide hazard, toxic effect, dietary ratio, frequency of pesticide use, presence of highly exposed populations and residue levels. In pears, eight high-risk pesticides were ranked in descending order as omethoate, carbofuran, isocarbophos, difenoconazole, chlorpyrifos, fenpyroximate, methomyl and flusilazole. In another study [43], a range of pesticides were detected in ‘Huangguan’ pear samples from a cultivation base in Wuwei, Gansu Province, China. These included carbofuran, chlorpyrifos, dichlorvos, omethoate, parathion, difenoconazole, triazophos and chlorpyrifos-methyl, with carbofuran exhibiting the highest detection rate at 67.0%, followed by chlorpyrifos at 33.0%.

It should be noted that several pesticides discussed previously (e.g., chlorpyrifos, dichlorvos, carbendazim, prochloraz, carbofuran, methomyl, etc.) have been banned, not approved or not renewed in some nations, particularly the EU, owing to mounting evidence of their toxicity and risks to human health and ecosystems [44]. Their presence in agricultural products, including pears, can be attributed to the following three key factors: (1) unauthorized application in agricultural practice due to limited alternatives; (2) metabolites or degradation products of approved pesticides; and (3) regulatory disparities in global trade, where exporting countries still permit their use [19]. This highlights the urgency of implementing continuous monitoring programs and promoting global harmonization of pesticide regulations to safeguard food safety and mitigate trade disputes.

**Figure 2:**
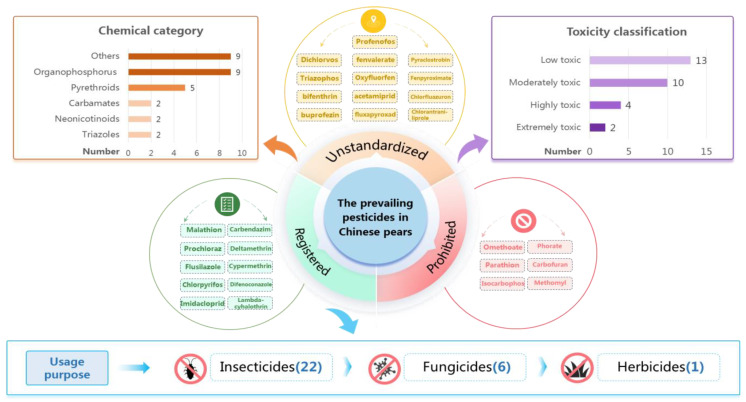
The prevailing pesticides in Chinese pears [22,23,24,26,27,28,34,35,36,37,38,39,41,42,43].

Figure 2 summarizes the pesticides detected in pears from China in recent years, including those with residues exceeding the MRLs. The data demonstrate the presence of 29 distinct residual pesticides in Chinese pears, where insecticides are predominant, accounting for 75.9% of all detections. Chemically, these pesticides mainly belong to the organophosphates and pyrethroids, and exhibit low to moderate toxicity. It is noteworthy that the majority (65.5%) of these pesticides are unregistered, indicating potential cases of illegal use of pesticides during pear production in China. According to China’s market supervision and monitoring regulations, agricultural products containing unregistered pesticides are prohibited for sale as substandard products; therefore, strengthening regulatory measures is crucial to mitigate the risk of exposure to consumers.

## 3. Dissipation of Pesticide in Pears

Numerous studies have investigated the dissipation of pesticides in pears. These studies typically involve regular sampling of field-grown pears after pesticide application, followed by quantitative analysis to determine residue concentrations. Subsequently, statistical methods are applied to develop mathematical models that describe the dissipation kinetics of pesticides. This well-established protocol is also extensively applied to examine pesticide dissipation in various other fruit crops, such as grapes and citrus [8,28], providing critical data for determining appropriate pre-harvest intervals (PHIs) to ensure that pesticide residue levels in pears and other fruits remain below MRLs [45]. Following field application, pesticides dissipate due to various factors, such as their physicochemical properties, formulation, dosage, application methods and environmental conditions (e.g., temperature, humidity, rainfall and light intensity) [46].

The dosage and application method of pesticides significantly influence their deposition in fruits, such as pears, which subsequently affects their dissipation dynamics. A study by Schusterova et al. [47] investigated the fate of 17 pesticides in pear orchards in the Czech Republic from 2020 to 2022. The study revealed substantial variability in the dissipation rates of these pesticides, with half-lives (i.e., the time required for the pesticide concentration to reduce to half of its initial level) ranging from 3.3 to 54.1 days. This variability was attributed to differences in pesticide properties, formulations, application frequencies and quantities. Of the pesticides tested, pyrimethanil showed the highest dissipation rate, whereas acetamiprid exhibited the lowest. These findings provide a scientific foundation for optimizing pesticide use in pear cultivation within temperate climates. In another study, Wang et al. [48] evaluated the dissipation of chlorpyrifos (48% emulsifiable concentrate) in ‘Whangkeumbae’ pear during the fruit inflating stage, employing varying dosages (D1: regular dose diluted 2000 times; D2: recommended dose diluted 1000 times; D3: double the recommended diluted 500 times) and bagging treatments. The results showed that higher dosages led to greater initial chlorpyrifos deposition, following the order D3 > D2 > D1. Bagging treatments also significantly influenced deposition, with the following sequence: pre-bagging spray > no bagging > post-bagging spray. Conversely, the dissipation rate followed an inverse trend: no bagging > post-bagging > pre-bagging. This phenomenon is likely due to chlorpyrifos’ photosensitivity, where exposure to light and elevated temperatures accelerate dissipation. Bagging, by shielding pears from light and wind, consequently reduces the dissipation rate [34]. Further elucidating the impact of application methods, Wu et al. [49] investigated the dissipation and residue levels of acephate and its metabolite methamidophos in nectarine, juicy peach and pear fruits using three application techniques: direct spray, bagged spray and root irrigation. In pear fruits, direct spraying resulted in significantly higher concentrations of both compounds compared to bagged spray and root irrigation. Acephate dissipation under direct spray was determined to follow the first-order kinetics, with a half-life of 8.5 days. Notably, 20 days post-application, the concentrations of both compounds under all spraying methods fell below China’s MRLs (500 μg/kg for acephate and 50 μg/kg for methamidophos), affirming the safety of acephate use in pear cultivation.

Geographical location also plays a critical role in pesticide dissipation. Lan et al. [50] conducted a two-year field study on clothianidin (20% suspension concentrate) dissipation in pears across three Chinese provinces (Shandong, Anhui and Hebei). The dissipation rates varied significantly, with Shandong exhibiting the highest rate, followed by Anhui and Hebei, corresponding to mean half-lives of 13.5, 14.1 and 15.6 days, respectively. Similarly, Kabir et al. [51] observed the dissipation of cyenopyrafen in Asian pears cultivated in Naju and Gochang, South Korea. The dissipation rate in Naju was markedly slower than in Gochang, with a half-life difference of 4.6 days, attributed to variations in temperature, light intensity and cultivar characteristics between the two locations. Environmental factors, particularly temperature, have an influential impact on pesticide dissipation. Elevated temperatures enhance pesticide evaporation by increasing vapor pressure and volatility, thereby accelerating processes such as solubility changes, toxicity alterations and half-life reduction. Conversely, colder environments decelerate dissipation processes like volatilization, photodecomposition and microbial degradation. Fang et al. [52] explored the dissipation behavior and residue distribution of prochloraz, pyraclostrobin and tebuconazole in Dangshan Su pears stored at 25 °C and 2 °C. At 2 °C, the half-lives ranged from 99.0 to 346.6 days, whereas at 25 °C, they were significantly shorter (8.8–13.9 days). Among these fungicides, tebuconazole, with the lowest residue concentration in pear pulp (maximum 0.226 mg/kg) and the longest half-life (≥231.0 days), was identified as the most suitable fungicide for preserving Dangshan Su pears during storage. However, the metabolic capability of pear for fungicides diminishes at lower storage temperatures, increasing the risk of prolonged exposure. A study by Tang et al. [53] demonstrated that the half-lives of thiophanate-methyl, tebuconazole, pyraclostrobin and difenoconazole in pears increased 2.9–8.2-fold at 4 °C compared to 25 °C, suggesting that these fungicides may persist in pears under low-temperature storage, thereby elevating exposure risks. This is corroborated by findings that some commercially available pears contained preservative levels exceeding China’s MRLs.

In conclusion, the dissipation of pesticides in pears is influenced by a complex interplay of factors including application methods, dosages, geographical location and environmental conditions, particularly temperature. Understanding these dynamics is crucial for ensuring the safe and effective use of pesticides in pear cultivation. Table 1 summarizes the findings from studies on the dissipation of various pesticides in pears. It is evident that, apart from chlorpyrifos and carbendazim, the majority of pesticides exhibit low initial deposits in pears and are classified as easily degradable, with a half-life of less than 30 days [54]. The persistence of a pesticide is typically described by its half-life, which is calculated as ln2/*k*. The relationship between pesticide residue concentration and time elapsed since application is commonly described using the first-order kinetic model: $C_{t}=C_{0}e^{-kt}$, where *C_t_* represents the concentration of the pesticide at time *t* (days), *C*_0_ denotes the initial concentration at time *t* = 0 (days) and *k* is the first-order rate constant (day^−1^) [31]. Based on these findings, it is recommended that, during pear production, pesticides with slower dissipation rates be applied initially, followed by those with faster dissipation rates, depending on the species of pests and diseases and their occurrence patterns. This strategy will facilitate a reduction in pesticide residue concentrations in pears, thereby enhancing consumer safety.

## 4. Pesticide Residue Detection in Pears

### 4.1. Sample Preparation

Pesticide levels in pears and other fruit samples are generally low and often fall below the detection limits of many analytical instruments; thus, it is crucial to extract, isolate and concentrate these compounds from such sample matrices to minimize matrix effects and enhance the selectivity and sensitivity of the analytical procedure, thereby ensuring reliable and accurate results. Given that pesticides have a wide range of types and chemical structures with diverse physicochemical properties, such as solubility and polarity, the selection of an appropriate solvent is a key factor in developing a successful extraction process. In many cases, acetonitrile [26,28,67,68] is frequently the solvent of choice due to its numerous benefits, including excellent solubility for most pesticides, higher recovery rates and a significant reduction in the co-extraction of matrix components, which simplifies subsequent cleanup steps. Currently, various techniques have been exploited for the extraction of PRs in fruit matrices, including pears, with vortex-assisted extraction [24,25,67,68] and ultrasonic extraction [69,70,71] being the most frequently utilized. Among these, vortex-assisted extraction is often preferred because of its simplicity, speed, cost-effectiveness, low energy consumption and high recovery rates for target analytes. As an example, a study by Cheng et al. [72] utilized this method to extract 15 organophosphorus pesticides from various F&Vs, including pear, apple, cucumber, tomato and cabbage, using acetonitrile as the solvent. The study achieved good extraction yields, with recoveries above 70% after just 10 min of extraction at a sample/solvent ratio of 1:1. However, crude extracts from pear and other fruit matrices often contain co-extracted substances, such as sugars, vitamins and organic acids, which can interfere with the accurate quantification of pesticides. Thus, a cleanup step is essential to remove these undesirable compounds and obtain higher-quality extracts for precise analysis.

**Figure 3:**
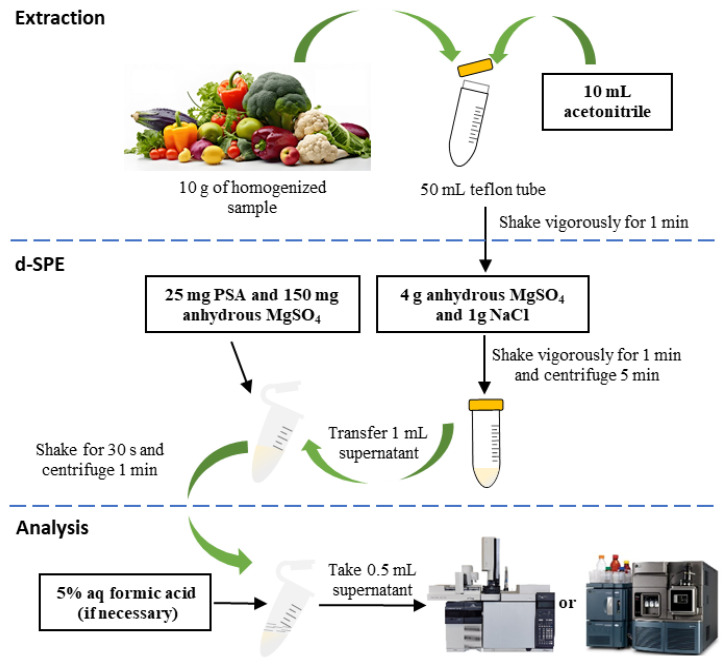
Schematic diagram of a typical QuEChERS analysis procedure applied for pesticide residue detection in F&Vs.

Solid-phase sorption-based methods, such as solid phase extraction (SPE) and dispersive SPE (d-SPE), are currently the primary techniques applied for purifying extracts from fruit matrices, including pears, in pesticide residue analyses. It is well established that sorbents act significantly in these methods due to their adsorption performance towards pesticides and their impact on the method’s selectivity and sensitivity. In SPE, unwanted impurities (e.g., chlorophyll) are either adsorbed onto the sorbent material, or the analytes of interest are adsorbed while the impurities are eluted. The concentrated analytes are then removed from the column for analysis. d-SPE, derived from the Quick, Easy, Cheap, Effective, Rugged and Safe (QuEChERS) method (Figure 3), which involves directly adding the sorbent to the sample extract, followed by dispersion to allow full interaction with the sample matrix in a short time. Upon completion of the dispersion process, the sorbent, containing the analytes on its surface, is separated using mechanical means such as centrifugation or filtration. This method offers several advantages, including short treatment time, adaptability, low cost and simplicity. Very recently, Liu [73] compared these two methods and found that d-SPE outperformed SPE in determining 21 PRs in 8 F&Vs, including pear, tomato, cucumber, carrot, lettuce, orange, peach and watermelon. d-SPE demonstrated greater flexibility in the cleanup process, as it allowed the use of different sorbents tailored to the properties of various matrices, and achieved higher recovery rates. As highlighted above, the suitable sorbent materials are the guarantee for achieving high cleanup performance and recovery rates. To date, a variety of sorbents with diverse chemistries, including NH_2_, octadecylsilane (C_18_), primary secondary amine (PSA) and graphitized carbon black (GCB), have been utilized due to their effectiveness, cost-efficiency and easy availability. In laboratory practice, selecting the appropriate sorbent, or a combination thereof, is key to successful SPE or d-SPE, and it primarily depends on the nature of the sample matrix, the pesticides being analyzed and the specific analytical objectives. Although SPE demonstrates efficacy in purifying or isolating PRs from diverse food matrices, including pears, this technique exhibits several drawbacks. One is that the cartridges may become clogged by suspended particulate matter in samples, potentially reducing recovery rates due to undesirable sorbent–analyte interactions. Furthermore, the non-reusability of SPE columns contributes to increased experimental costs. Similarly, d-SPE presents several disadvantages. For instance, in some cases, it may fail to adequately remove interferents from crude sample extracts, thereby inducing matrix effects during analytical procedures. In contrast to conventional SPE, d-SPE suffers from poor automation compatibility, where manual processing steps (e.g., vortexing and centrifugation) frequently result in inter-user variability.

Sample preparation techniques commonly used for pesticide residue detection in pears, along with the type of sorbent and extraction solvent, have been summarized in Table 2.

**Figure 4:**
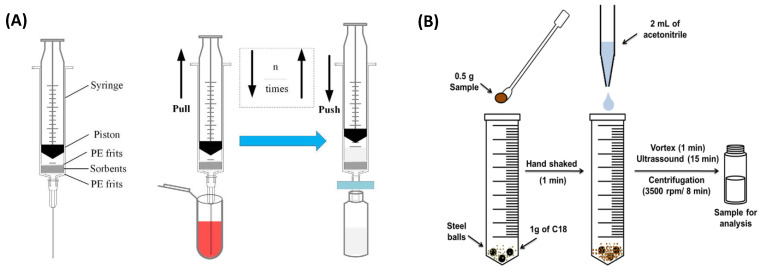
(**A**) Schematic diagram of m-PFC syringe and the cleanup procedure. (**B**) Scheme of the BiT-MSPD procedure.

In addition to the aforementioned techniques, several innovative approaches have emerged as alternative laboratory methods for the analysis of PRs in pears. Recently, Zhang et al. [78] proposed an array-thin film micro-extraction (aTFME) method for the analysis of 13 PRs in agricultural products, including pear, tea and cabbage. In this method, a polyacrylonitrile–hydrophile lipophile balance (PAN-HLB) film was prepared as the extraction material and directly immersed in the sample solution to adsorb target pesticides. The adsorbed pesticides were subsequently desorbed using a mixture of acetonitrile/methanol/water (17/2/1, *v*/*v*/*v)* for quantification, achieving recovery rates over 70% and relative standard deviations (RSDs) below 12.0%. This technique is straightforward to operate, combining extraction, isolation and purification in a single step, and offers high throughput, enabling the processing of up to 96 samples per batch. Moreover, the aTFME film can be cleaned with methanol for reuse, making it an environmentally friendly and cost-effective option. In a separate study, Meng et al. [79] applied a multi-plug filtration cleanup (m-PFC) technique following UE extraction to purify extracts from pear and 11 other F&Vs for determining 234 PRs. As illustrated in Figure 4A, the m-PFC procedure involves a 5 mL syringe housing two polyethylene sieve plates with sorbents, such as PSA and multiwalled carbon nanotubes (MWCNTs), packed between them. The extract is slowly filtered through the sorbents by alternately pulling and pushing the piston several times, followed by instrumental analysis. This method effectively removes interferents, such as pigments, providing extracts with acceptable purity and accurate pesticide quantification, with recovery rates ranging from 72.8% to 122.4%. Magnetic dispersive µ-solid-phase extraction (MD-µSPE), a miniaturized form of SPE, has garnered significant attention for its rapid, sustainable and high-throughput preconcentration and removal of contaminants from food matrices. This technique addresses the limitations of conventional SPE by omitting time-consuming steps such as centrifugation and filtration. The development of advanced magnetic sorbents has been pivotal to its success. As an illustrative example, Shirani et al. [80] developed an MD-µSPE technique for the simultaneous separation and preconcentration of 15 trace-level pesticides in apple and pear samples. In this method, sulfonated melamine-modified NiFe_2_O_4_ nanoparticles (SM NiFe_2_O_4_ NPs) were prepared and employed as the magnetic sorbent, achieving enrichment factors ranging from 291.5 to 397.5. Recovery assays validated the method‘s applicability, with satisfactory recoveries ranging from 92.5% to 98.9% and RSDs ≤ 4.3% for both pear and apple matrices. Furthermore, Kemmerich et al. [81] introduced a novel technique called balls-in-tube matrix solid-phase dispersion (BiT-MSPD) for analyzing 133 PRs in pear, apple, peach and plum. As depicted in Figure 4B, the BiT-MSPD method allows all sample preparation steps to be conducted within a closed extraction tube using steel balls, with C_18_ as the sorbent material and acetonitrile as the elution solvent. Compared to conventional MSPD, BiT-MSPD is faster and more efficient, as extraction and cleanup occur within the same tube, eliminating the need for transfers to cartridges or additional cleanup steps. This technique enables rapid extraction (25 min) with minimal solvent consumption (2 mL) and achieves high recovery rates (72–113%) for the analytes. A key advantage of this method is its potential for full automation of the sample preparation process. As previously noted, despite exhibiting significant advantages (e.g., simplicity and convenience), these emerging methods have yet to achieve widespread adoption for the detection of PRs in pears compared to well-established techniques like SPE, d-SPE, or QuEChERS.

### 4.2. Detection Techniques

Owing to the diverse types and structures of pesticides, selecting an appropriate methodology for detecting PRs in pears, as in other food matrices, requires careful consideration of multiple factors, such as the nature of the target pesticides, specific detection requirements and available laboratory conditions. In recent years, chromatography and mass spectrometry have emerged as the most extensively used approaches for this purpose. Each technique offers distinct advantages and limitations, with key factors such as sensitivity, selectivity and sample preparation influencing the choice of method. Table 2 provides a summary of recently developed techniques within this domain for determining PRs in diverse pear samples.

#### 4.2.1. Chromatography and Mass Spectrometry

Chromatographic methods, including gas chromatography (GC) and high-performance liquid chromatography (HPLC), are among the earliest techniques commonly employed for the quantification of PRs in pears. Despite their cost-effectiveness, user-friendliness and ease of instrument maintenance, these methods require rigorous sample preparation and exhibit limited sensitivity and identification capabilities for pesticides, leading to a decline in their utilization in recent times.

To address this concern, there has been a shift toward more sensitive and reliable methodologies, such as tandem mass spectrometry (MS/MS) and high-resolution mass spectrometry (HRMS), which have gained popularity for routine analysis due to advancements in analytical instrumentation. Specifically, triple quadrupole MS (QqQ-MS) and quadrupole-time-of-flight MS (QTOF-MS), combined with GC, HPLC, or ultra-high performance liquid chromatography (U(H)PLC), have emerged as leading technologies for pesticide residue analysis in pears (Table 2). In QqQ-MS, ions are separated in the first mass analyzer, and specific precursor ions are selected and fragmented to produce product ions, which are then detected by the second mass analyzer. This approach has significantly expanded the range of detectable pesticides, enabling the identification and quantification of hundreds of pesticides in a short time through multiple reaction monitoring (MRM) of characteristic precursor and product ions, and relevant determinations have been described in recent studies. As an example, utilizing a GC-QqQ-MS system, 143 pesticides, including organophosphorus, organochlorine, pyrethroids, carbamate and their metabolites, were separated and detected within 16 min. When coupled with QuEChERS-based extraction using PSA and C_18_ as d-SPE sorbents, this method successfully determined these pesticides in seven agricultural products, including pear, apple, agaric, cucumber, potato, spinach and tomato. All pesticides demonstrated high recovery rates (≥84.1%) and precision (RSDs ≤ 10.4%), with limits of detection (LODs) and quantification (LOQs) of 2.0 μg/kg and 5.0 μg/kg, respectively. Notably, the entire analytical process was completed within 30 min, highlighting the method’s efficiency [82]. Further demonstrating its utility, Kemmerich et al. [83] developed a UHPLC-QqQ-MS method for the multi-residue analysis of 170 pesticides in pear samples following QuEChERS extraction without cleanup. The method achieved LOQs of 2.5–10 µg/kg, with recovery rates between 70 and 120% and RSDs ≤ 20%. This study revealed significant concerns about PRs in pears from Brazil, as 21 pesticides were quantified at concentrations ranging from 3.3 to 1427 μg/kg. In some countries, such as China, QqQ-MS coupled with GC or HPLC is incorporated into national standard methods for the multi-residue analysis of pesticides and their metabolites in foods of plant origin, including pears, providing robust technical support for monitoring PRs [84,85]. However, the application of QqQ-MS is limited by its low resolution, which compromises quantification accuracy and makes it less appropriate for screening unknown pesticides.

QTOF-MS, with its high resolution and accuracy, has emerged as a viable alternative. This technique is a powerful tool for both quantitative analysis and the identification of unknown compounds based on accurate masses, fragment ions and retention times in full-scan mode. For instance, Munaretto et al. [86] developed a multiclass screening and rapid quantitative method utilizing HPLC-QTOF-MS in full-scan mode to determine 152 PRs in pear, apple and grape. The QTOF-MS detection, based on protonated molecular ions and/or adducts with mass accuracy, provided reliable results. Recovery rates for over 130 pesticides were satisfactory (66–122%), with RSDs ≤ 28% and LODs between 10 and 40 µg/kg. PRs were identified in all five pear and apple samples as well as in four grape samples purchased from local supermarkets in Santa Maria, Rio Grande do Sul, Brazil. In pears, four pesticides (i.e., carbendazim, thiabendazole, thiacloprid and thiophanate methyl) were detected at levels ranging from 12 to 177 µg/kg, none of which exceeded MRLs set by the EU. In another study [87], QTOF-MS coupled with atmospheric pressure GC was applied to screen 104 pesticides and other organic contaminants in pears, achieving LODs as low as 0.02 μg/kg. This technique was also successfully applied to various other F&Vs, including apple, cucumber, tomato, cabbage, leek and grape, yielding satisfactory results. More advances in mass analyzers have also been reported. For example, Gkountouras et al. [88] combined Linear Trap Quadrupole/Orbitrap (LTQ/Orbitrap) HRMS with UHPLC for the targeted analysis of 30 pesticide compounds in pears and 81 other fruits. The method achieved satisfactory recoveries (76.8–108%) with RSDs < 13.4%, LODs < 10 μg/kg for most analytes and a combined measurement uncertainty < 50%, indicating its suitability for measuring low pesticide concentrations. In pear samples from Greece, three pesticides (cyclostrobin, tebuconazole and myclobutanil) were identified at concentrations varying from 3.2 to 80.6 μg/kg. Furthermore, the technique included a suspect screening of 355 pesticides and their transformation products (TPs), tentatively identifying 71 compounds, which included 22 previously unlisted pesticides and TPs. However, it is important to highlight that these advanced methods are highly specialized and costly, making them inaccessible to most routine laboratories.

#### 4.2.2. Other Techniques

Several other techniques have been developed for the detection of PRs in pears and other sample matrices, such as surface-enhanced Raman spectroscopy (SERS) and enzyme linked immunosorbent assay (ELISA). These methods are highly appreciated for their increased sensitivity, exceptional specificity and cost-effectiveness. Recently, Wang et al. [89] developed a SERS-based aptasensor for the ultrasensitive and interference-free detection of chlorpyrifos in pear, cucumber and river water samples. The aptasensor utilized gold nanoparticles coated with Prussian blue (Au@PB NPs) conjugated with aptamers as SERS probes and magnetic nanoparticles (MNPs) combined with the complementary aptamer (cApt) as capture probes. The Raman report exhibited a sole, narrow and intense signal at 2160 cm^−1^, endowing the aptasensor with unique anti-interference capabilities. The method achieved a low LOD of 0.066 μg/L and recovery rates in the range of 85.4–108.0% with RSDs ≤ 7.7%, which were consistent with those obtained by the HPLC-QqQ-MS method, thereby confirming the method’s reliability. ELISA determines pesticides through the principle of antigen–antibody interaction coupled with enzyme-catalyzed colorimetric changes. For small molecules such as pesticides, indirect competitive ELISA (ic-ELISA) is usually developed for their determination in various food matrices. For instance, Yu et al. [90] established an ic-ELISA method utilizing a specific monoclonal antibody against imidacloprid for its sensitive detection in pear, rice and cabbage, achieving a low LOD of 0.06 μg/L, which is more sensitive than the most reported methods. The recovery rates for spiked samples varied from 83.6% to 112.7%, with a coefficient of variation (CV) < 11.53%. These results demonstrated a strong correlation between the developed ELISA and a commercial kit (R^2^ = 0.9531). In another study [91], an ic-ELISA method based on a broad-spectrum polyclonal antibody against organophosphorus pesticides was developed for the sensitive detection of methyl parathion and triazophos in pears, with LODs of 1.39 and 1.94  μg/L, respectively. To realize ultrasensitive detection of paraquat in pear and cabbage samples, Zhang et al. [92] introduced a biotin-streptavidin ELISA (BA-ELISA) method using a biotinylated nanobody (BiotinNb2-12) as a recognition element, combined with a biotin-horseradish peroxidase-labeled streptavidin (polyHRP-SA) affinity recognition signal system. Samples spiked with paraquat recovered above 94.5% with CV less than 18%. Compared to traditional ic-ELISA, the BA-ELISA method significantly reduced antibody consumption by 8-fold while improving sensitivity by 85-fold, achieving an impressive LOD of 0.00058 μg/L.

In a separate study, Jiang et al. [93] investigated PRs in fruits, including pear, carrot, kiwifruit and banana, utilizing 2-(diethexyphosphoryl) acetic acid as a common template molecule and Fe_3_O_4_@SiO_2_ as support material to prepare a superparamagnetic core/shell molecularly imprinting polymer (MIP), (Fe_3_O_4_@SiO_2_@MIP), which exhibits multiple recognition sites and increased adsorption capacity. Using Fe_3_O_4_@SiO_2_@MIP as a biomimetic antibody and quantum dots as a label, they developed a biomimetic fluorescence immunoassay method for the determination of methyl parathion, chlorpyrifos and trichlorfon. The assay demonstrated low LODs (0.21–0.44 μg/L), good recoveries (73.1–119.3%) and precision (RSDs ≤ 13.3%). All analyzed samples were found to contain the targeted pesticides, with concentrations ranging from 0.015 ± 0.002 to 0.307 ± 0.041 mg/kg.

Similarly, despite their demonstrated advantages (e.g., sensitivity and specificity), most of these established techniques remain confined to laboratory-scale applications without achieving widespread implementation in routine practice.

## 5. Future Trends

In recent years, substantial research progress has been made regarding PRs in pears, particularly in their monitoring, dissipation behavior and detection methodologies, yielding significant findings that propel advancements in pear quality and safety research. However, as a result of this review, it is clear that the data on regular monitoring of PRs in pears, dissipation kinetics (especially degradation data for high-risk pesticides like omethoate, fenpyroximate and methomyl) and practical implementation of emerging detection technologies are insufficient. It is imperative that future research be intensified in these areas. Furthermore, studies should focus on developing high-efficiency and low-risk pesticides specifically designed for pear pests and diseases. This aims to maximize pesticide efficacy with minimal application dosages, thereby reducing the accumulation of PRs. Moreover, emphasis should be placed on elucidating the dissipation mechanisms of PRs in pears and developing decontamination technologies or products to further mitigate their residual impacts. Continuous risk monitoring of pears is also crucial to assess the prevalence of PRs, especially the high-risk ones, which will assist in diminishing potential human exposure. Lastly, there must be a focus on the development of portable and rapid detection devices for on-site and real-time monitoring of PRs in various pears. It is hoped that the gaps in knowledge will soon be filled.

## 6. Conclusions

Pears are globally consumed fruits, and the presence of PRs in pears is a worldwide concern. This paper reviews existing research on PRs in pears, with a particular focus on their current status, dissipation pattern and detection methods. The findings demonstrate the widespread occurrence of PRs in pears across different regions, with frequent detection of multiple pesticides, including a significant proportion that are unapproved for use on pears. These results underscore the need for enhanced regulatory measures to mitigate consumer exposure risks. PRs degrade naturally over time, with dissipation rates and half-lives influenced by pesticide properties, application methods and environmental conditions. To minimize residue levels, early application of slower-degrading pesticides is recommended, followed by faster-dissipating alternatives, based on pest occurrence patterns. Various analytical approaches have been developed for PR detection and quantification, including modern sample preparation techniques (e.g., SPE, d-SPE) and sensitive detection methods (e.g., QqQ-MS coupled with GC or LC), each offering unique advantages and limitations. Consequently, method selection in routine monitoring should involve careful consideration of analytical performance characteristics, specific testing objectives and available laboratory resources. This review provides an introductory overview for researchers new to the field.

## Figures and Tables

**Table 1:** Dissipation pattern of different pesticides in pears.

Pesticide	Dosage, a.i.	Initial Deposit (mg/kg)	Dissipation		Half-Life (Day)	Comment (Trial Time and Location)	Ref.
			Kinetic Equation	Correlation Coefficient			
45% chlorpyrifos EC	600 mg/kg	2.692	*C_t_* = 1.5758e^−0.166*t*^	−0.98	4.2	2019, Anhui, Shandong and Heibei, China	[55]
25 g/L lambda-cyhalothrin EC	50 mg/kg	0.241	*C_t_* = 0.2619e^−0.096*t*^	−0.99	7.1		
10% imidacloprid SP	100 mg/kg	0.181	*C_t_* = 0.1558e^−0.056*t*^	−0.97	12.2		
50% carbendazim WP	2000 mg/kg	3.732	*C_t_* = 3.9849e^−0.057*t*^	−0.91	11.9		
480 g/L chlorpyrifos EC	450 mg/kg	4.68	*C_t_* = 4.1289e^−0.154*t*^	−0.98	4.4	2018	[56]
10% imidacloprid SP	30 mg/kg	0.12	*C_t_* = 0.1075e^−0.056*t*^	−0.96	12.2		
22.4% spirotetramat SC	90 mg/kg	0.044	*C_t_* = 0.0383e^−0.052*t*^	−0.98	13.1		
10% difenoconazole WDG	75 mg/kg	0.082	*C_t_* = 0.0586e^−0.066t^	−0.97	10.3		
0.3% matrine EC	0.27 g/m^2^	0.6633	*C_t_* = 0.4352e^−0.1418*t*^	−0.9806	4.89	Tianjing, China	[57]
		0.9140	*C_t_* = 0.4394e^−0.1761*t*^	−0.9608	3.94	Anhui, China	
24% fenbuconazole SC	144 mg/kg	0.6101	*C_t_* = 0.4889e^−0.073*t*^	−0.9711	9.5	2017, Heibei, China	[58]
		0.6692	*C_t_* = 0.5421e^−0.057*t*^	−0.9905	12.2	2017, Liaoning, China	
2.5% lambda-cyhalothrin EW	18.75 g/hm^2^	0.159	*C_t_* = 0.127e^−0.03*t*^	−0.9616	23.1	2016, Jinan, China	[59]
		1.050	*C_t_* = 0.948e^−0.09*t*^	−0.9939	7.7	2016, Taiyuan, China	
		0.424	*C_t_* = 0.278e^−0.07*t*^	−0.9478	9.9	2016, Hangzhou, China	
15% imibenconazole WP	75 mg/L	0.23	*C_t_* = 0.9461e^−0.042*t*^	−0.8859	16.5	2019, Yunnan, China	[60]
		0.15	*C_t_* = 0.3097e^−0.041*t*^	−0.9385	16.9	2019, Tianjing, China	
10% flusilazole EW	75 mg/kg	0.223	*C_t_* = 0.1547e^−0.079*t*^	−0.9763	8.83	2019, Shandong, China	[61]
40% myclobutanil SC	75 mg/kg	1.310	*C_t_* = 0.4875e^−0.048*t*^	−0.9669	14.44		
250 g/L tebuconazole EW	187.5 mg/kg	0.581	*C_t_* = 0.3720e^−0.148*t*^	−0.9517	4.70		
22.4% spirotetramat SC	112 mg/kg	0.086	*C_t_* = 0.0825e^−0.056*t*^	—	12.4	Hebei, China	[62]
50% fenitrothion EC	0.075 mL/m^2^	1.59	*C_t_* = 1.1704e^−0.226*t*^	−0.9936	3.07	2020, Zhejiang, China	[63]
250 g/Lpyraclostrobin SC	50 g/kg	0.466	*C_t_* = 0.4053e^−0.07*t*^	−0.9855	9.9	2020, Anhui, Shandong and Gnasu, China	[64]
10% bistrifluron SC	5 mL/20 L	0.29	*C_t_* = 0.3191e^−0.068*t*^	−0.9474	10.19	Naju, Republic of Korea	[65]
25% spinetoram WDG	0.3 kg/hm^2^	0.51	*C_t_* = 0.51e^−0.321*t*^	−0.9913	2.17	Kula, Serbia	[66]

**Table 2:** A summary of recently developed techniques for determining PRs in diverse pear samples.

Sample	Analytes	Sample Pretreatment	Instrumental Techniques	Instrumental Details	Analytical Performance	Ref.
Pear	34 pesticides	Vortex-assisted extraction with acetonitrile containing 1% acetic acid, purification by d-SPE using PSA as a sorbent	GC-MS/MS	HP-5MS column (15 m × 0.25 mm i.d., 0.25 µm); programmed temperature; splitless injection; inlet, ion source and transfer line temperature at 280 °C, 230 °C and 280 °C, respectively	Recoveries: 83.3–109.4% RSDs: 1.3–10.8% LOQs: 5.0 μg/kg	[24]
Pear	22 pesticides	Homogenization extraction with acetonitrile, without cleanup	UPLC-MS/MS	ReproSil 100 C_18_ column (25 cm × 2.1 mm i.d., 5 μm) at 35 °C with a gradient mobile phase of methanol and water containing 0.1% formic acid; positive electrospray ionization (ESI^+^); MRM.	Recoveries: 71.4–106.7% RSDs: 0.7–9.9% LODs: 0.9–4.6 μg/kg LOQs: 3.0–15.4 μg/kg	[26]
Pear	21 organophosphorus pesticides	Homogenization extraction with acetonitrile, purification by d-SPE using PSA as a sorbent	GC-MS	DB-5MS column (30 m × 0.25 mm i.d., 0.25 µm); programmed temperature; splitless injection; inlet, ion source and transfer line temperature at 280 °C, 230 °C and 280 °C, respectively	Recoveries: 85.4–100.4% RSDs: 1.9–6.8% LODs: 0.2–2.6 μg/kg	[28]
pear	31 pesticides	Vortex-assisted extraction with acetonitrile containing 1% acetic acid, purification by d-SPE using PSA and C_18_ as sorbents	HPLC-MS/MS	C_18_ column (10 cm × 2.1 mm i.d., 1.8 µm) at 30 °C with a gradient mobile phase of acetonitrile and water containing 0.1% formic acid; ESI^+^ at 350 °C; MRM.	Recoveries: 75.0–111.5% RSDs: 0.9–6.7% LODs: 0.25–25 μg/kg	[43]
Apple-pear	19 organochlorine pesticides	Ultrasonic extraction with acetonitrile, purification by an SPE cartridge using NH_2_ as a sorbent and eluting with methanol/dichloromethane (1:19, *v*/*v*)	GC-MS	TG-5MS capillary column (30 m × 0.25 mm i.d., 0.25 µm); programmed temperature; splitless injection; inlet and ion source temperature at 290 °C and 280 °C, respectively.	Recoveries: 86.1–108.9% RSDs: 4.0–9.5% LODs: 3.0–6.0 μg/kg LOQs: 10–20 μg/kg	[69]
Pear	Myclobutanil, diniconazole, epoxiconazole, methoxychlor	Ultrasonic extraction with acetonitrile, purification by d-SPE using PSA and GCB as sorbents	GC-MS/MS	DB-5MS column (30 m × 0.25 mm i.d., 0.25 µm); programmed temperature; splitless injection; inlet, transfer line and ion source temperature at 250 °C, 250 °C and 200 °C, respectively	Recoveries: 80–111% RSDs: 0.8–1.2% LOQs: 10.0 μg/kg	[70]
Pear and tomato	9 pesticides	Ultrasonic extraction with acetonitrile, without cleanup	UPLC-MS/MS	BEH C_18_ column (5 cm × 2.1 mm i.d., 1.7 μm) at 35 °C with a gradient mobile phase of acetonitrile and water containing 0.1% formic acid; positive electrospray ionization (ESI^+^) at 110 °C; MRM.	Recoveries: 61.7–116.5% RSDs: 0.7–18.9% LODs: 0.1–4.0 μg/kg LOQs: 10 μg/kg	[71]
Pear, grape and apple	15 pesticides and adjuvants	Vortex-assisted extraction with acetonitrile, purification by an SPE cartridge using NH_2_ as a sorbent and eluting with methanol/dichloromethane (5:95, *v*/*v*)	UPLC-MS/MS	Shim-pack XR-ODS column (7.5 cm × 2.0 mm i.d., 1.6 μm) at 40 °C with a gradient mobile phase of methanol and water containing 2 mmol/L ammonium acetate and 0.05% formic acid; ESI^+^/ESI; MRM.	Recoveries: 80–112% RSDs: 5.5–16% LOQs: 5-10 μg/kg	[74]
Grains and vegetables including pears	Metamifop	Vortex-assisted extraction with *n*-hexane and acetonitrile/water (5:5, *v*/*v*) containing 1% acetic acid, purification by d-SPE using PSA and polystyrene/ divinylbenzene as sorbents	HPLC-MS/MS	JADE-PAK CB-C_18_ column (10 cm × 2.1 mm i.d., 3.0 μm) at 30 °C with a gradient mobile phase of acetonitrile and water containing 0.1% formic acid; ESI+; MRM.	Recoveries: 63.9–113.7% RSDs: 1.0–22.2% LODs: 0.2–0.3 μg/kg LOQs: 0.6–1.0 μg/kg	[75]
Pear	Polyoxin B and oxine-copper	Vortex-assisted extraction with methanol and water containing 1% acetic acid (5:95, *v*/*v*) containing 1% acetic acid, purification by d-SPE using PSA as a sorbent	UPLC-MS/MS	SB-Aq column (10 cm × 3.0 mm i.d., 1.8 μm) at 35 °C with a gradient mobile phase of methanol and water containing 0.1% formic acid; ESI^+^ at 150 °C; MRM.	Recoveries: 78–99% RSDs ≤ 5.2% LOQs: 5–10 μg/kg	[76]
Pear, grape, jujube and apricot	99 pesticides	Ultrasonic extraction with acetonitrile, purification by d-SPE using PSA and C_18_ as sorbents	GC-MS/MS	TG-5MS column (30 m × 0.25 mm i.d., 0.25 µm); programmed temperature; splitless injection; inlet, ion source and transfer line temperature at 260 °C, 280 °C and 280 °C, respectively.	Recoveries: 70–120% RSDs: 0.3–20% LOQs: 10–25 μg/kg	[77]

## Data Availability

No new data were created or analyzed in this study. Data sharing is not applicable to this article.

## References

[1] Xylia P., Chrysargyris A., Ahmed Z.F.R., Tzortzakis N. (2021). Application of rosemary and eucalyptus essential oils and their main component on the preservation of apple and pear fruits. Horticulturae.

[2] Reiland H., Slavin J. (2015). Systematic review of pears and health. Nutr. Today.

[3] Hong S.Y., Lansky E., Kang S.S., Yang M.H. (2021). A review of pears (*Pyrus* spp.), ancient functional food for modern times. BMC Complement. Med. Ther..

[4] USDA Foreign Agricultural Service Fresh Apples, Grapes, and Pears: World Markets and Trade. https://www.fas.usda.gov/sites/default/files/2024-12/fruit.pdf.

[5] Wang W.H., Wang G.P., Tian L.M., Li X.G., Lv X.L., Zhang Y.X., Zhang J.H., Cao Y.F. (2019). Fruit scientific research in New China in the past 70 years: Pear. J. Fruit Sci..

[6] Food and Agriculture Organization of the United Nations Database Crops and Livestock Products. https://www.fao.org/faostat/en/#data/TCL.

[7] Wang X.C., Yang M.N., Huang D.S. (2022). Research progress on pear diseases and insect pests. South China Agric..

[8] Liu F.Q., Ming L., Zhao Y.C., Sun W.B. (2023). Progress in the occurrence and control of pear fire blight in China. Deciduous Fruits.

[9] Guo P., Li M., Gao M.X., Guo J.L., Ma A.H. (2024). Current status of pesticide registration for the control of grapholitha molesta in China. Pestic. Sci. Admin..

[10] Husain M., Rathore J.P., Sharma A., Raja J., Qadri I., Wani A.B.W. (2018). Description and management strategies of important pests of pear: A review. J. Entomol. Zool. Stud..

[11] (2021). National Food Safety Standard—Maximum Residue Limits for Pesticides in Food.

[12] European Commission EU Pesticides Database. https://ec.europa.eu/food/plant/pesticides/eu-pesticides-database/start/screen/mrls.

[13] Codex Alimentarius Commission Codex Pesticides Residues in Food Online Database. https://www.fao.org/fao-who-codexalimentarius/codex-texts/dbs/pestres/en/.

[14] The Japanese Ministry of Health, Labour and Welfare Maximum Residue Limits (MRLs) List of Agricultural Chemicals in Foods. http://db.ffcr.or.jp/front/food_group_detail?id=9200.

[15] USDA Foreign Agricultural Service Pesticide Data Program Annual Summary, Calendar Year 2021. https://www.ams.usda.gov/sites/default/files/media/2021PDPAnnualSummary.pdf.

[16] USDA Foreign Agricultural Service Pesticide Data Program Annual Summary, Calendar Year 2022. https://www.ams.usda.gov/sites/default/files/media/2022PDPSummary.pdf.

[17] Eissa F., Zidan N.E.-H., Sebaei A.S., Mohamed M.E.B. (2024). Pesticide residues in fruits and vegetables: Analysis and risk assessment of EU RASFF notifications between 1999 and 2022. J. Food Compos. Anal..

[18] Jardim A.N.O., Caldas E.D. (2024). Pesticide residues in food of plant origin commercialized in Brazil from 2010 to 2020—An update from the two national monitoring programs. Food Control.

[19] Carrasco Cabrera L., Di Piazza G., Medina Pastor P., EFSA (European Food Safety Authority) (2022). The 2020 European Union report on pesticide residues in food. EFSA J..

[20] Carrasco Cabrera L., Di Piazza G., Dujardin B., Medina Pastor P., EFSA (European Food Safety Authority) (2023). The 2021 European Union report on pesticide residues in food. EFSA J..

[21] Carrasco Cabrera L., Di Piazza G., Dujardin B., Marchese E., Medina Pastor P., EFSA (European Food Safety Authority) (2024). The 2022 European Union report on pesticide residues in food. EFSA J..

[22] Li H., Chang Q.Y., Bai R.B., Lv X.C., Cao T.L., Shen S.G., Liang S.X., Pang G.F. (2021). Simultaneous determination and risk assessment of highly toxic pesticides in the market-sold vegetables and fruits in China: A 4-year investigational study. Ecotox. Environ. Safe.

[23] Chen M.H., Zeng Y., Ouyang W.M. (2023). Current status and analysis of pesticide residues in fruits and vegetables grown in Huili region plant bases. Anal. Test. Technol. Instr..

[24] Qian X., Yang Q.R., Zheng Z.S., Chen Y.D. (2018). Simultaneous determination of 34 kinds of pesticide residues in pears by gas chromatography-tandem mass spectrometry. J. Food Saf. Qual..

[25] Zhao Y., Wang W., Yin D.Y., Liang X.C., Qiao H.O. (2023). Investigation of pesticide residues in commercialized fruits and risk assessment of dietary intake in Shaanxi Province from 2018 to 2021. Chin. J. Food Hygi..

[26] Chi M.Y., Chen Z.L., Guo C.Y., Ding R.Y., Fang L.P., Zhang W.J., Mao J.S., Li H.D. (2019). Simultaneous determination of 22 kinds of pesticide residues in pear by ultra-high performance liquid chromatography-tandem mass spectrometry. J. Food Saf. Qual..

[27] Lu Q., Li H.D., Ding R.Y., Guo C.Y., Fang L.P., Zhang W.J., Mao J.S., Chen Z.L. (2019). Determination of seven pyrethroid pesticide residues in pear by GC-MS with QuEChERS. Agrochemicals.

[28] Zhang Y., Li H.D., Ding R.Y., Guo C.Y., Mao J.S., Chen Z.L. (2019). Simultaneous determination of 21 organophosphorus pesticide residues in pears by QuEChERS-GC-MS. Food Ind..

[29] Hakme E., Herrmann S.S., Poulsen M.E. (2020). Processing factors of pesticide residues in biscuits and their relation to the physicochemical properties of pesticides. Food Addit. Contam. A.

[30] Albaseer S.S. (2019). Factors controlling the fate of pyrethroids residues during post-harvest processing of raw agricultural crops: An overview. Food Chem..

[31] Farha W., Abd El-Aty A.M., Rahman M.M., Jeong J.H., Shin H.-C., Wang J., Shim J.-H. (2018). Analytical approach, dissipation pattern and risk assessment of pesticide residue in green leafy vegetables: A comprehensive review. Biomed. Chromatogr..

[32] Hakme E., Hajeb P., Herrmann S.S., Poulsen M.E. (2024). Processing factors of pesticide residues in cereal grain fractions. Food Control.

[33] Ahmadi S., Khazaei S., Mehri F. (2024). Determination of pesticide residues in fruits: A systematic review and meta-analyses. J. Food Compos. Anal..

[34] Gao Y.Q., Li Q., Fang N. (2021). Analysis of the monitoring results of 39 pesticide residues in the main fruits of Daxing district, Beijing from 2017 to 2019. J. Food Saf. Qual..

[35] Liu Y.H., Bei K., Zheng W.R., Yu G.G., Sun C.X. (2023). Pesticide residues risk assessment and quality evaluation of four characteristic fruits in Zhejiang Province, China. Front. Environ. Sci..

[36] Qin D.P., Yan F., Yang Q.Z., Huang S.Y., Chen G., Jing L.Q. (2022). Investigation of 208 pesticide residues in fruits in Chongqing in 2021. Environ. Chem..

[37] Wang S.W., Wang K.X., Zhou H.X., Sun Y.Z. (2024). Investigation and analysis of pesticides use risk and safety in Shandong province. China Plant Protect..

[38] Yang Y.C., Rebuji S.B., He M., Liu X. (2023). Analysis of the monitoring results of pesticide residues in agricultural products of Yanbian prefecture, Jilin Province from 2021 to 2022. Agric. Technol..

[39] Zhang J., Ma Q.Q., Wang X., Yuan P., Su Y.H., Zhang R.J. (2021). Investigation on pesticides residues in common fruits and vegetables in Zhengzhou city. Henan J. Prev. Med..

[40] EFSA (European Food Safety Authority) (2025). Review of the methodology used for the assessment of the short-term (acute) dietary exposure to pesticide residues in food (IESTI methodology). EFSA J..

[41] Zeng J., Qiao X.W. (2023). A brief analysis of pesticide residues exceeding maximum residue limits in vegetables and fruits in China. Chin. J. Pestic. Sci..

[42] Lan F., Wang Z.X., Lu Z.Q., Yao J., Jiang W., Liu X., Wang X.C., Zhou X.X., Liu C.D. (2017). Risk ranking of pesticide residues in apples and pears in Shandong Province. Plant Prot..

[43] Duan A.L., Guo F.C., Zhang N.J. (2022). Determination of 31 pesticide residues in Huangguan pear by HPLC-MS/MS. China Food Saf..

[44] European Commission EU Pesticides Database. https://ec.europa.eu/food/plant/pesticides/eu-pesticides-database/start/screen/active-substances.

[45] Wang Z.L., Song J., Zhao J.F., Zhai C.Y. (2023). Research progress on the degradation characteristics and mathematical models of pesticide residues in fruit trees. Jiangsu Agric. Sci..

[46] Farha W., Abd El-Aty A.M., Rahman M.M., Shin H.-C., Shim J.-H. (2016). An overview on common aspects influencing the dissipation pattern of pesticides: A review. Environ. Monit. Assess..

[47] Schusterova D., Horska T., Skalsky M., Stara J., Ourednickova J., Uttl L., Kocourek V., Hajslova J. (2024). Three-year monitoring study of pesticide dissipation in pears. J. Food Compos. Anal..

[48] Wang J.T., Fang Y.J., Yan X.H., Wang H., Yang B.D., Wang X.W., Zhang Z.Y. (2018). Residual dynamics of chlorpyrifos during the fruit inflating stage of Whangkeumbae pear. J. Food Saf. Qual..

[49] Wu X.W., Xue J.Y., Pan D.D., Jin L.J., Shi T.Z., Cheng X.X., Li Q.X., Hua R.M. (2017). Dissipation and residue of acephate and its metabolite metamidophos in peach and pear under field conditions. Int. J. Environ. Res..

[50] Lan F., Liu X., Li X.L., Zhou X.X., Liu C.D., Wang Z.X., Lu Z.Q., Jiang W. (2018). Residues and dissipation of clothianidin in pears. Chin. J. Pestic. Sci..

[51] Kabir M.H., El-Aty A.M.A., Kim S.-W., Rahman M.M., Chung H.S., Lee H.S., Shin H.-C., Shim J.-H. (2017). Decline pattern and risk assessment of cyenopyrafen in different varieties of Asian pear using liquid chromatography and tandem mass spectrometry. Food Sci. Biotechnol..

[52] Fang Q.K., Wu R.F., Hu G.X., Lai A.P., Wu K.X., Zhang L.W., Feng J.J., Cao H.Q. (2020). Dissipation behavior, residue distribution and risk assessment of three fungicides in pears. J. Sci. Food. Agric..

[53] Tang Y.F., Hu K.K., Li X.M., Liu C.G., Xu Y.H., Zhang Z.X., Wu X.W. (2022). Dissipation dynamics and dietary risk assessment of four fungicides as preservatives in pear. Agriculture.

[54] Liang Z., Mahmoud Abdelshafy A., Luo Z.S., Belwal T., Lin X.Y., Xu Y.Q., Wang L., Yang M.Y., Qi M., Dong Y.Y. (2022). Occurrence, detection, and dissipation of pesticide residue in plant-derived foodstuff: A state-of-the-art review. Food Chem..

[55] Mao J.S., Chen Z.L., Li H.D., Guo C.Y., Ding R.Y., Zhang W.J., Yan M.M. (2021). Residues and dissipation dynamics of 4 pesticides in pear. Agrochemicals.

[56] Mao J.S., Chen Z.L., Li H.D., Zhang W.J., Ding R.Y., Fang L.P., Guo C.Y. (2019). Residues and dissipation dynamics of chlorpyrifos, imidacloprid, spirotetramat and its motablites, difenoconazole in pear. Chin. J. Pestic. Sci..

[57] Lu L.N., Shen Y., Gao M.J., Zhong J.F., Lu F., Zheng Z.T., Zhang Z.Y. (2024). Residue and dissipation of matrine in pear and soil. Zhejiang Agric. Sci..

[58] Li Z.X., Yan Z., Nie J.Y., Chen Y., Shen Y.M. (2018). Study on dissipation dynamics of fenbuconazole in pear and acute risk assessment of dietary intake. Qual. Saf. Agro-Prod..

[59] Du H.X., Li H.D., Guan S., Chen Z.L. (2018). Study on residue and degradation of cyhalothrin in pear and soil. Shandong Agric. Sci..

[60] Tang S.H., Huang L., Dai X.F., Deng Y.S., Pu E.T., Li W.X., Zhang X.Y. (2021). Residual behavior and dietary risk assessment of imibenconazole in pears. Agrochemicals.

[61] Gao M.J., Shen Y., Lu L.N., Zhong J.F., Zheng Z.T., Zhang Z.Y. (2023). Residues and dissipation characteristics of three fungicides in pear and soil. Chin. Agric. Sci. Bull..

[62] Qian X., Zheng Z.S., Chen Y.D., Zhang S.J., Guan J.F., Fan L.X., Zhao X.D., Qian M.Y. (2019). Residues and dissipation dynamics of spirotetramat and its metabolites in pear and soil. Chin. J. Pestic. Sci..

[63] Liu Y.H., Xu M.F., Teng M.Y., Pang Y.J., Sun C.X. (2022). Residue dissipation and safe medication recommendations of imidacloprid in honey pear. Zhejiang Agric. Sci..

[64] Zhu M.Q., Mao J.S., Chen Z.L., Li H.D., Guo C.Y., Zhang W.J. (2023). Residue and dissipation dynamics of pyraclostrobin in pear. J. Anhui Agric. Sci..

[65] Truong L.T.B., Kim S.W., El-Aty A.M.A., Kabir H., Rahman M., Choi J.H., Shin H.C., Kwon C.H., Lee K.B., Yoon H.J. (2016). Various extraction methods for detection of bistrifluron residues in Asian pear using high-performance liquid chromatography: Application to dissipation patterns under open-field conditions. Biomed. Chromatogr..

[66] Šunjka D., Lazić S., Vuković S., Alavanja A., Nađ Đ., Mitrić S. (2021). Residue and dissipation dynamic of spinetoram insecticide in pear fruits. Plant Protect. Sci..

[67] Tankiewicz M. (2019). Determination of selected priority pesticides in high water fruits and vegetables by modified QuEChERS and GC-ECD with GC-MS/MS confirmation. Molecules.

[68] Zhou Q.Z., Liu Z.Q., Liu F.M., Guo Y.G., Li X.H. (2021). Determination of desmedipham residue in 21 foods by HPLC-MS/MS combined with a modified QuEChERS and mixed-mode SPE clean-up method. J. Food Compos. Anal..

[69] Yao Y.H., Bai L.L., Wu L.P., Wu X.Z. (2019). Determination of 19 kinds of organchlorine pesticide residues in apple-pear by using gas chromatography-mass spectrometry coupled with solid-phase extraction. Food Mach..

[70] Wang F.H., Hao J., Chen M., Li Z. (2024). Determination of 4 pesticide residues in pear by gas chromatography-triple quadrupole mass spectrometry method. China Food Saf. Mag..

[71] Zhang H.Y. (2018). Determination of nine pesticide residues in tomatoes and pears by UPLC-MS/MS. Agrochemicals.

[72] Cheng Z.P., Dong F.S., Xu J., Liu X.G., Wu X.H., Chen Z.L., Pan X.L., Gan J., Zheng Y.Q. (2017). Simultaneous determination of organophosphorus pesticides in fruits and vegetables using atmospheric pressure gas chromatography quadrupole-time-of-flight mass spectrometry. Food Chem..

[73] Liu X.B. (2024). Comparative study on the simultaneous determination of 21 pesticide residues in 8 fruits and vegetables by QuEChERS and SPE pre-treatment combined with GC-MS/MS. Cereal. Oil..

[74] Lan F., Li X.L., Duan X.N., Jiang W., Zang H.W., Liu C.D., Zhou X.X., Lu Z.Q., Wang Z.X., Hua Z.K. (2019). Determination of 15 high-risk pesticide and adjuvant residues in fruits from USA by solid phase extraction-ultra performance liquid chromatography-tandem mass spectrometry. Chin. J. Pestic. Sci..

[75] Lei H.Q., Su Y.Z., Asika X.R.F.H., Li Y.M., Li F., Zhou J. (2024). Determination of metamifop residues in fruits, vegetables and grains by dual-liquid extraction QuEChERS-high performance liquid chromatography-tandem mass spectrometry. Anal. Instrum..

[76] Hu C.L., Wang Q.S., Zhang L., Fu Y., Xu F., Chen H.X., Wu Y.L. (2021). Determination of polyoxin B and oxine-copper in pear by ultra performance liquid chromatography-tandem mass spectrometry. J. Food Saf. Qual..

[77] Zhang H.Y., Li Y.M., Hua P. (2023). Determination of 99 pesticide residues in four fruit products by gas chromatography-tandem mass spectrometry. Chin. J. Pestic. Sci..

[78] Zhang F., Wang J., Yan M.M., Zhu C., Feng J.J., Tan T.Y., Liu J.H., Li T.F. (2022). Determination of 13 kinds of pesticide residues in agricultural products by array-thin film micro-extraction coupled with high performance liquid chromatography- tandem mass spectrometry. J. Food Saf. Qual..

[79] Meng Z.J., Huang Y.X., Di P.Y., Zhao L.M., Niu L.S., Fan S.F., Li Q., Zhang Y. (2020). Rapid Screening of 234 pesticide residues in vegetables and fruits by multi-plug filtration cleanup method combined with gas chromatography-quadrupole time of flight mass spectrometry. Food Sci..

[80] Shirani M., Ansari F., Shabanian M., Wagenknecht U., Salamat Q., Faraji M., Basij M., Adeli M. (2024). NiFe2O4 nanoparticles grafted sulfonated melamine for rapid magnetic dispersive µ-solid phase extraction of pesticides in apple and pear samples: Greenness evaluation. Microchem. J..

[81] Kemmerich M., Demarco M., Bernardi G., Prestes O.D., Adaime M.B., Zanella R. (2020). Balls-in-tube matrix solid phase dispersion (BiT-MSPD): An innovative and simplified technique for multiresidue determination of pesticides in fruit samples. J. Chromatogr. A.

[82] Du L.J., Tian J.R., Xue Y., Zhang Y.F., Chang T.Y. (2021). Rapid detection of 143 pesticide residues in agricultural products by QuEChERS and gas chromatography-mass spectrometry. China Port Sci. Technol..

[83] Kemmerich M., Bernardi G., Prestes O.D., Adaime M.B., Zanella R. (2018). Comprehensive method validation for the determination of 170 pesticide residues in pear employing modified QuEChERS without clean-up and ultra-high performance liquid chromatography coupled to tandem mass spectrometry. Food Anal. Methods.

[84] (2018). National Food Safety Standard—Determination of 208 Pesticides and Metabolitesresidues in Foods of Plant Origin—Gas Chromatography-Tandem Mass Spectrometry Method.

[85] (2021). National Food Safety Standard—Determination of 331 Pesticides and Metabolitesresidues in Foods of Plant Origin—Liquid Chromatography-Tandem Mass Spectrometry Method.

[86] Munaretto J.S., Viera M.D.S., Martins M.L., Adaime M.B., Zanella R. (2016). Quantitative multiclass pesticide residue analysis in apple, pear, and grape by modified QuEChERS and liquid chromatography coupled to high-resolution mass spectrometry. J. AOAC Int..

[87] Cheng Z.P., Zhang X.Z., Geng X., Organtini K.L., Dong F.S., Xu J., Liu X.G., Wu X.H., Zheng Y.Q. (2018). A target screening method for detection of organic pollutants in fruits and vegetables by atmospheric pressure gas chromatography quadrupole-time-of-flight mass spectrometry combined with informatics platform. J. Chromatogr. A.

[88] Gkountouras D., Boti V., Albanis T. (2024). High resolution mass spectrometry targeted analysis and suspect screening of pesticide residues in fruit samples and assessment of dietary exposure. Environ. Pollut..

[89] Wang H., Chen Z.L., Zhu C., Du H.X., Mao J.S., Qin H.W., She Y.X., Yan M.M. (2023). An interference-free SERS-based aptasensor for chlorpyrifos detection. Anal. Chim. Acta.

[90] Yu X.D., Li Y.S., Si Z.Z., Liu Y., Guan N.Y., Zhang S.L., Zha Y.H., Zhou Y. (2019). Development of an indirect competitive ELISA for the detection of imidacloprid. J. Yangzhou Univ..

[91] Yue Y.D., Chen J.Y., Zhang M.J., Yin Y.G., Dong Y.Y. (2022). Determination of organophosphorus pesticides in vegetables and fruit by an indirect competitive enzyme-linked immunosorbent assay (ic-ELISA) and a lateral-flow immunochromatographic (LFIC) strip assay. Anal. Lett..

[92] Zhang Y.Y., Yang J.Y., Zeng D.P., Xu Z.L., Wang H., Tian Y.X., Sun Y.M., Shen Y.D. (2024). Ultrasensitive immunoassay for paraquat residues in fruits and vegetables based on biotinylated nanobodies. J. Instrum. Anal..

[93] Jiang M.D., He J.B., Gong J.J., Gao H.J., Xu Z.X. (2019). Development of a quantum dot-labelled biomimetic fluorescence immunoassay for the simultaneous determination of three organophosphorus pesticide residues in agricultural products. Food Agric. Immunol..

